# The Prevalence of Serum Immunoglobulin G Antibody to Chlamydia Trachomatis in Subfertile Women Presenting at the University of Port Harcourt Teaching Hospital, Nigeria

**Published:** 2011-06

**Authors:** Israel Jeremiah, Ola Okike, Chris Akani

**Affiliations:** *Department of Obstetrics and Gynaecology, University of Port Harcourt Teaching Hospital, Port Harcourt, Nigeria*

**Keywords:** chlamydia trachomatis, immunoglobulin G, subfertility

## Abstract

**Objectives:**

This study was undertaken to assess the prevalence of IgG antibody to *Chlamydia trachomatis* in subfertile patients at the University of Port Harcourt Teaching Hospital and to determine associated factors between this and infertility.

**Study design:**

This case controlled study was conducted among 100 women presenting for infertility consultation at the University of Port Harcourt Teaching Hospital. One hundred women with normal intrauterine pregnancies attending the antenatal clinic were used as controls. A questionnaire was used to obtain information on their socio-demographic data, sexual and obstetric history administered to them. 2mls of venous blood was collected, labelled and sent to the laboratory. The presence of IgG antibody to Chlamydia trachomatis was determined. Hysterosalpingography was performed on all infertile women to assess tubal patency. Data management was with SPSS 15.0 for Windows^®^ statistical software.

**Results:**

The mean age of the subjects was 30 ± 3.1 years, median parity 0.5 and average life time sexual partner 3.7 ± 2.8. All the participants in the study were married. 62% of subjects had tertiary education. The *Chlamydia trachomatis* IgG antibody prevalence in the subfertile population was 74% and 51% in the control group, *P*<0.001. Tubal occlusion occurred in 58 (78.4%) of cases positive for chlamydia antibody. Pelvic inflammatory disease and mucopurulent discharge were the most common presentating symptoms among Chlamydia antigen positive infertile women, *P*<0.001. There was an association between subfertility and the number of life time sexual partners. There was an association between subfertility and non usage of condoms.

**Conclusion:**

The prevalence IgG antibody to *Chlamydia trachomatis* was significantly higher in women with subfertility compared to women with proven fertility. There was a strong association between Chlamydia antibody positivity and tubal occlusion. In a resource-poor country such as Nigeria, enzyme immunosorbent assay for chlamydial IgG antibodies may be substituted for HSG for the detection of tubal occlusion.

## INTRODUCTION

*Chlamydia trachomatis* is an obligate intracellular bacterium that belongs to the order Chlamydiales and the family Chlamydiaceae (1). It is currently recognized as the most common sexually transmitted pathogen (2). The World Health Organization (WHO) estimated that there were 15.4 million new cases in the sub-Saharan Africa in 1995 with an increase to 15.85 million in 1999 (3). It is also recognized as the most prevalent and damaging of all sexually transmitted diseases (STD) (3).

Clinical history is not reliable in making a diagnosis of *Chlamydial* infection (4, 5) and WHO estimates that 70-75% of women infected with *C. trachomatis* are symptom-free (1). The sequel of undetected and thus untreated infections like acute salpingitis and pelvic inflammatory disease lead to significant morbidity and importantly to infertility. Infertility due to *C. trachomatis* represents a preventable type of infertility if detected early (6).

The detection of current *C. trachomatis* infection utilizes gene amplification technique such as Polymerase chain reaction (PCR) and Ligase chain reaction (LCR) which have now replaced cell culture technique. Past infection with *C. trachomatis* can be demonstrated by IgG antibody (7, 8).

Since the association between *C. trachomatis* IgG antibody in serum and tubal factor infertility was noted some workers have used Chlamydia antibody testing (CAT) as a screening test for tubal pathology in the infertility for work-up (8-10). Following *C. trachomatis* infection, usually, in adolescence, a decade or two may elapse before women present with subfertility (1). The demonstration of IgG antibody in subfertile women may be useful as a marker for a subgroup of women at increased risk of tubal pathology. Chlamydia antibody testing (CAT) is considered a useful tool in the management of infertility and some workers have demonstrated comparable predictive value to hysterosalpingogram (HSG) in tubal factor disease (10). Additionally, it has the advantage of being less painful, non-invasive, simple and cost effective (11).

The reported negative predictive value (NPV) of CAT in subfertile women ranges from 75-90% depending on the test used and the laboratory (12-15). A major concern however, is the effect of false positive results on patients’ management and personal lives (8-15).

There is paucity of data on the association between *C. trachomatis* and infertility in Nigeria. Possibly as a result this, there is no policy on screening of the disease in subfertile patients. Thus this study aims to evaluate *Chlamydia trachomatis* infection in women presenting with sub-fertility.

## MATERIALS AND METHODS

This case controlled study was conducted among 100 consecutive women presenting for infertility consultation at the University of Port Harcourt Teaching Hospital over a six month period between 1st September 2008 and 28th February 2009. Only those who had normal ovulatory tests and whose husbands had normal results on a semenogram were recruited into the study. One hundred women with normal intrauterine pregnancies attending the antenatal clinic were randomly selected and used as controls excluding women with history of subfertility prior to achieving pregnancy. All the participants were informed of the study and all agreed to participate. All the subjects had detailed history and physical examinations. A questionnaire to elicit their socio-demographic data, sexual behaviours and previous history of pelvic infections and obstetric performance was then administered.

For serology testing 2 mls of venous blood was collected from each participant, labelled and sent to the laboratory. The serum was separated, frozen and stored and IgG antibody to *Chlamydia trachomatis* was tested according to the manufactures instructions using enzyme immunoassay (EIA) with the ImmunoComb kit by Orgenics^®^. Seven samples were not properly stored and those subjects were excluded from the study. Hysterosalpingography was performed on all subfertile women to assess tubal patency.

The data collected were coded and entered into a computer using SPSS 15.0 for Windows^®^ statistical software which was also used for data analysis. Results are presented as means with standard deviations, rates and proportions in tables and figures. Student t-test was carried out where necessary. Cross tabulations and correlation analysis were performed to establish relationships among variables. Statistical significance was assumed at *P* values of ≤0.05.

The study was approved by the Institutional Ethics Committee of the University of Port Harcourt Teaching Hospital. A written informed consent was obtained from each patient.

## RESULTS

Table 1 shows that the socio-demographic characteristics of both the study group and the control group were similar. The mean age of subjects was 30 ± 3 years. Seventy percent of the subjects were in the age group from 26 to 35 years. The mean age of the control group was 29.2 ± 4.4 years. All the participants were married. Most were in monogamous marriage (study group 91%, control 92%). Sixty two (62%) of subjects had tertiary education and while 44% of control had tertiary education. The mean parity in the study population was 0.3 ± 0.7 with 77% being nulliparous. The mean parity in the control was 1.0 ± 1.3, with 51% being nulliparous.

**Table 1:** Socio-demographic characteristics

Characteristic	Study group	Control group	*p*-value

Age (Mean ± SD)	30 ± 3 years	29.2 ± 4.4 years	0.97
Parity (Mean ± SD)	0.3 ± 0.7	1.0 ± 1.3	0.052
Marital status			
Married	100	100	-
Single	0	0	-
Educational status			
No formal education	1	0	-
Primary	1	0	-
Secondary	36	46	0.35
Tertiary	62	54	0.55

**Figure 1:**
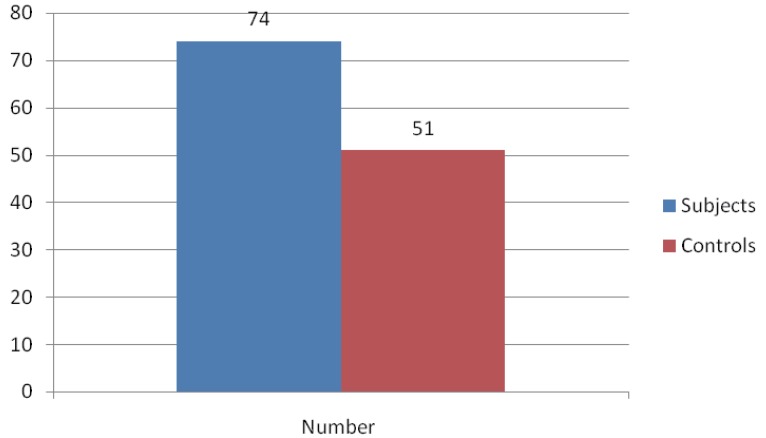
Chlamydia Ig G prevalence.

Among the study group, 74% had IgG antibody to *Chlamydia trachomatis* in their serum, while 51% of the control was positive for the antibody (Figure 1). This difference was statistically significant (*P*<0.001). Among the subfertile women who tested positive for IgG antibody to *C. trachomatis*, 58 (78.4%) had tubal occlusion.

**Figure 2:**
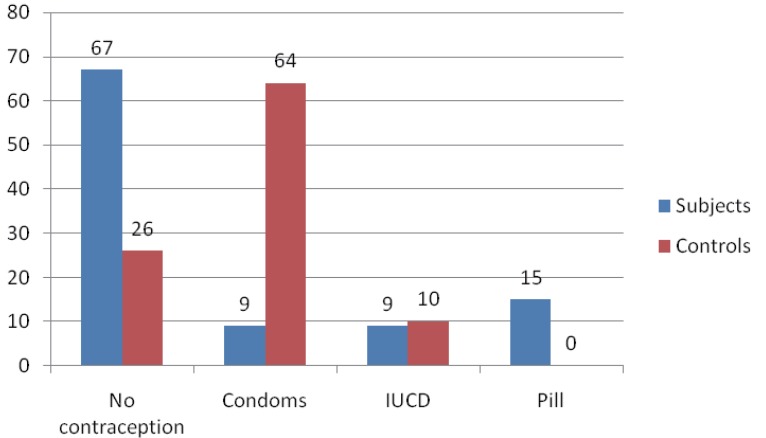
Contraceptive use.

The mean number of life-time sexual partners in the subjects and controls were 3.7 ± 2.8; and 1.8 ± 1.0 respectively. The difference was statistically significant at *P*=0.048. Figure 2 shows contraceptive use among both groups. Sixty seven percent of the study group had not used contraception. Among those who used contraception only 9 (9%) used condoms. This contrasts with the control group where 64 (64%) used condoms and only 24% had not used contraception. This difference in condom use was statistically significant (*P*<0.01).

The average number of induced abortions was similar in both groups. It was 1.92 ± 2 with a range of 0 to 9 in the subjects while in the control this was1.32 ± 1.7 with a range of 0 to 8. The average number of miscarriages in the subjects was 0.1 ± 0.3 while in the controls it was 0.340 ± 0.7.

Presenting symptoms of women with secondary infertility in relation to Chlamydia status are given in Table 2. Pelvic inflammatory disease and mucopurulent discharge were the most common presentations, *P*<0.001. Twelve women (16.2%) had previous miscarriages, while 6 (8.1%) had a history of previous ectopic pregnancy.

**Table 2:** Clinical profile of women with symptoms with secondary infertility in relation to Chlamydia positivity

Clinical presentation	No. of symptomatic cases (n=74)

Bleeding per vaginam	5
Mucopurulent discharge	51
PID	46
Previous miscarriage	12
Previous ectopic pregnancy	6
Chronic cervicitis	4
Dysuria	9

## DISCUSSION

The prevalence of Chlamydia trachomatis IgG antibody in this study was 74% in the subfertile group and 51% in the pregnant controls. This difference was statistically significant (*p*-value 0.001). Of the infertile women with positive Chlamydia antigen, 78.4% had tubal occlusion. This demonstrates the role played by Chlamydia in the causation of secondary infertility. Studies in Nigeria and other West African countries show prevalence from 23.4% to 65.8% in subfertile population, 17.3% in pregnant women and 12% in house wives (16-19). In a study in Northern Nigeria 38.3% infertile subjects with tubal disease and 13.3% family planning clients had current Chlamydia infection (20). Three quarters of women with STI in Europe have anti-chlamydial antibodies in their serum (21). An earlier study in Port Harcourt on the prevalence of active *C. trachomatis* infection in the age group 14-19years found a prevalence of 37.5% (22).

Based on these findings, the chlamydial antibody prevalence in this study was not unexpected. This is because antibody prevalence studies mirrors past infections in the women and there has been a trend towards increasing *C. trachomatis* infection world wide. The differences in the result can therefore be explained by age, socioeconomic background and sexual practices which have considerable influence on the prevalence of *C. trachomatis*.

Not surprisingly, 78.4% of the patients with positive results for IgG antibodies had tubal infertility, *P*<0.001. This finding supports a high correlation between Chlamydia antibody positivity and tubal blockage and suggests that in a resource-poor country such as Nigeria, EIA for chlamydial IgG antibodies can be substituted for HSG for detection of tubal occlusion. Other studies have reported similar results (13). Some studies have reported that *C. trachomatis* antibody testing is more accurate than HSG in predicting tubal factor infertility (23).

Nine percent and sixty four percent of the subjects and controls respectively had used condoms. This difference is significant, *P*<0.001. Some studies have found significant association between non-use of condom and *C. trachomatis* genital infection (24). This study did not determine consistency of use, however, it clearly demonstrates that sexually active women who did not use condoms were more likely to acquire chlamydial infection. The study group had statistically significant higher number of life time sexual partners suggesting that high risk sexual practices were a risk factor for Chlamydia trachomatis infection.

The risk of pregnancy loss was higher in the subjects when compared to the control. Symptoms of pelvic infection and STD did not differ in the two populations. This was the finding in a study in Northern Nigeria and may be due to the asymptomatic nature of Chlamydia trachomatis infection (20).

The limitation of the study included the fact that the test kit used for above study was qualitative, so results were either positive or negative for IgG antibody to chlamydia. A quantitative test that determines the titre level of the antibody may show a difference in titre range for the positive results in the controls and subjects. An antibody titre level could then be determined that correlate with tubal disease.

## CONCLUSIONS

The prevalence of *Chlamydia trachomatis* was higher in the study group with subfertility when compared to the pregnant controls. There was a strong association between Chlamydia antibody positivity and tubal occlusion. In a resource-poor country such as Nigeria, enzyme immunosorbent assay for chlamydial IgG antibodies may be substituted for HSG for the detection of tubal occlusion. Since infertility resulting from Chlamydia trachomatis is preventable, clinicians should be more aware of its deleterious effects even in asymptomatic women who will benefit from early screening and treatment. 
